# Potential risk of colonization of *Bulinus globosus* in the mainland of China under climate change

**DOI:** 10.1186/s40249-022-00980-2

**Published:** 2022-05-13

**Authors:** Xinyao Wang, Saleh Juma, Wei Li, Mchanga Suleman, Mtumweni Ali Muhsin, Jian He, Mingzhen He, Dacheng Xu, Jianfeng Zhang, Robert Bergquist, Kun Yang

**Affiliations:** 1 Jiangsu Institute of Parasitic Diseases, Wuxi, 214064 Jiangsu China; 2 Key Laboratory On Technology for Parasitic Disease Prevention and Control, Jiangsu Provincial Key Laboratory On the Molecular Biology of Parasites, Ministry of Health, Wuxi, 214064 Jiangsu China; 3 Ministry of Health of Zanzibar, P.O. Box 236, Zanzibar, United Republic of Tanzania; 4 Changzhou Center for Disease Control and Prevention, Changzhou, Jiangsu China; 5 Jintan Center for Disease Control and Prevention, Changzhou, Jiangsu China; 6 College of Medicine, Jiangnan University, Wuxi, 214122 Jiangsu China; 7 Ingerod, SE-454 94 Brastad, Sweden; 8 School of Public Health, Nanjing Medical University, Nanjing, China

**Keywords:** *Bulinus globosus*, Colonization, Potential distribution, Geographic information systems, Climate change, *Schistosoma haematobium*, *China*

## Abstract

**Background:**

*Bulinus globosus,* the main intermediate snail host of *Schistosoma haematobium*. The increased contacts between Africa and China could even lead to large-scale dissemination of *B. globosus* in China. Temperature is the key factor affecting fresh-water snail transmission. This study predicted potential risk of colonization of *B. globosus* in the mainland of China under climate change.

**Methods:**

We investigated minimum and maximum temperatures for *B. globosus* eggs, juveniles and adult snails kept under laboratory conditions to find the most suitable range by pinpointing the median effective temperatures (ET50). We also assessed the influence of temperature on spawning and estimated the accumulated temperature (AT). The average air temperatures between 1955 and 2019 in January and July, the coldest and hottest months in China, respectively, were collected from national meteorological monitoring stations and investigated in a geographic information system (GIS) using empirical Bayesian Kriging to evaluate the theoretical possibility for distribution of *B. globosus* in southern China based on temperature.

**Results:**

The effective minimum temperature (ET50_min_) for eggs, juveniles, adult snails and spawning were 8.5, 7.0, 7.0, 14.9 °C, respectively, with the corresponding maximum values (ET50_max_) of 36.6, 40.5, 40.2 and 38.1 °C. The AT was calculated at 712.1 ± 64.9 °C·d. In 1955, the potential *B. globosus* distribution would have had a northern boundary stretching from the coastal areas of Guangdong Province and Guangxi Autonomous Region to southern Yunnan Province. Since then, this line has gradually moved northward.

**Conclusions:**

Annual regeneration of *B. globosus* can be supported by the current climate conditions in the mainland of China, and a gradual expansion trend from south to north is shown in the study from 2015 to 2019. Thus, there is a potential risk of colonization of *B. globosus* in the mainland of China under climate change.

**Graphical Abstract:**

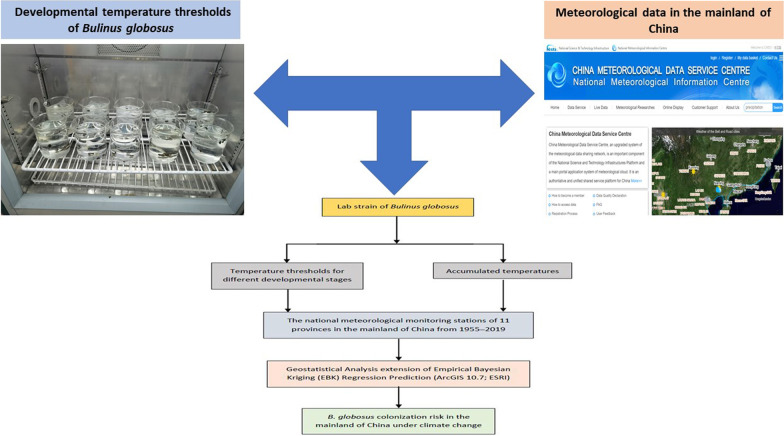

## Background

Schistosomiasis is a parasitic disease with a life cycle that involves an intermediate snail host. It endangers human health and affects social and economic development in 78 countries and regions in Africa, Asia, South America and the Middle East. About 230 million people are infected with more than 800 million at risk [1–3]. Five schistosome species: *Schistosoma japonicum*, *S. mansoni*, *S. haematobium*, *S. intercalatum* and *S. mekongi* and a few subgroups can parasitize humans, with the three former species being the most common [4]; *S. mansoni* is the only species in Latin America*,* while *S. mansoni* and *S. haematobium* dominate in Africa. *S. japonicum* is the only species in China and the Philippines and some minor pockets in Indonesia; while Cambodia and Laos share a limited endemic area of *S. mekongi* [5]. All species except *S. haematobium* cause intestinal symptoms, while the latter produces urogenital symptoms, including bladder cancer [6, 7]. No locally infected schistosomiasis haematobia cases exist in Southeast Asia but imported cases do, particularly in China [8].

*Bulinus* (Mollusca, Gastropoda, Pulmonata, Basommatophora, Planorbidae) is a hermaphroditic group of freshwater snails of about 30 species that all can transmit *S. haematobium* [9]. These snails are widely distributed in Africa and countries bordering the Indian and the Mediterranean Seas. According to the shape of the shell and some characteristics of the organism, four main groups of *Bulinus* species have been named: *B. africanus*, *B. forskalii*, *B. reticulatus* and the *B. truncatus/tropicus* complex. *B. globosus*, the main intermediate host of *S. haematobium,* belongs to the *B. africanus* group.

Temperature is the key factor governing the distribution of snails, because there are other main factors such as light that affecting snails [10, 11]. According to the 6^th^ Assessment Report (https://www.ipcc.ch/assessment-report/ar6/) of the Intergovernmental Panel on Climate Change (IPCC), the global warming trend is intensifying, which means that it might affect the *B. globosus* distribution sooner than previously thought, a fact that has attracted widespread attention from scholars around the world [12, 13]. China is the largest prevalent area of schistosomiasis japonica and, so far, no *S. haematobium* intermediate snail host has been found there [14]. However, with the global warming and the increasing frequency of international exchange, especially that related to advancing the “Belt and Road” initiative, trade between China and Africa has become grown, and many local African aquatic plants and snails can be brought back to China or purchased through the Internet. Many invasive species survive and multiply since individuals first kept at home in their aquariums only to later discard them in the natural environment. These developments have led to an increased risk of the introduction of *B. globosus* into China.

Empirical Bayesian Kriging (EBK) is a fast and reliable solution for both automatic and interactive data interpolation. It can be used for interpolation of very large datasets, which has resolved the drawbacks of classical geostatistical models [15]. In recent years, EBK has been applied in numerous applied research papers to predict changes in space and climate, for example Kutuzov [16], Nocco [17] and Nogueira [18]. In this research, we used the statistical software of Geostatistical Analysis extension of EBK Regression Prediction of ArcGIS 10.7 (ESRI) to generate maps both for indicating snail survival and for predicting the *B. globosus* colonization risk.

**Fig. 1:**
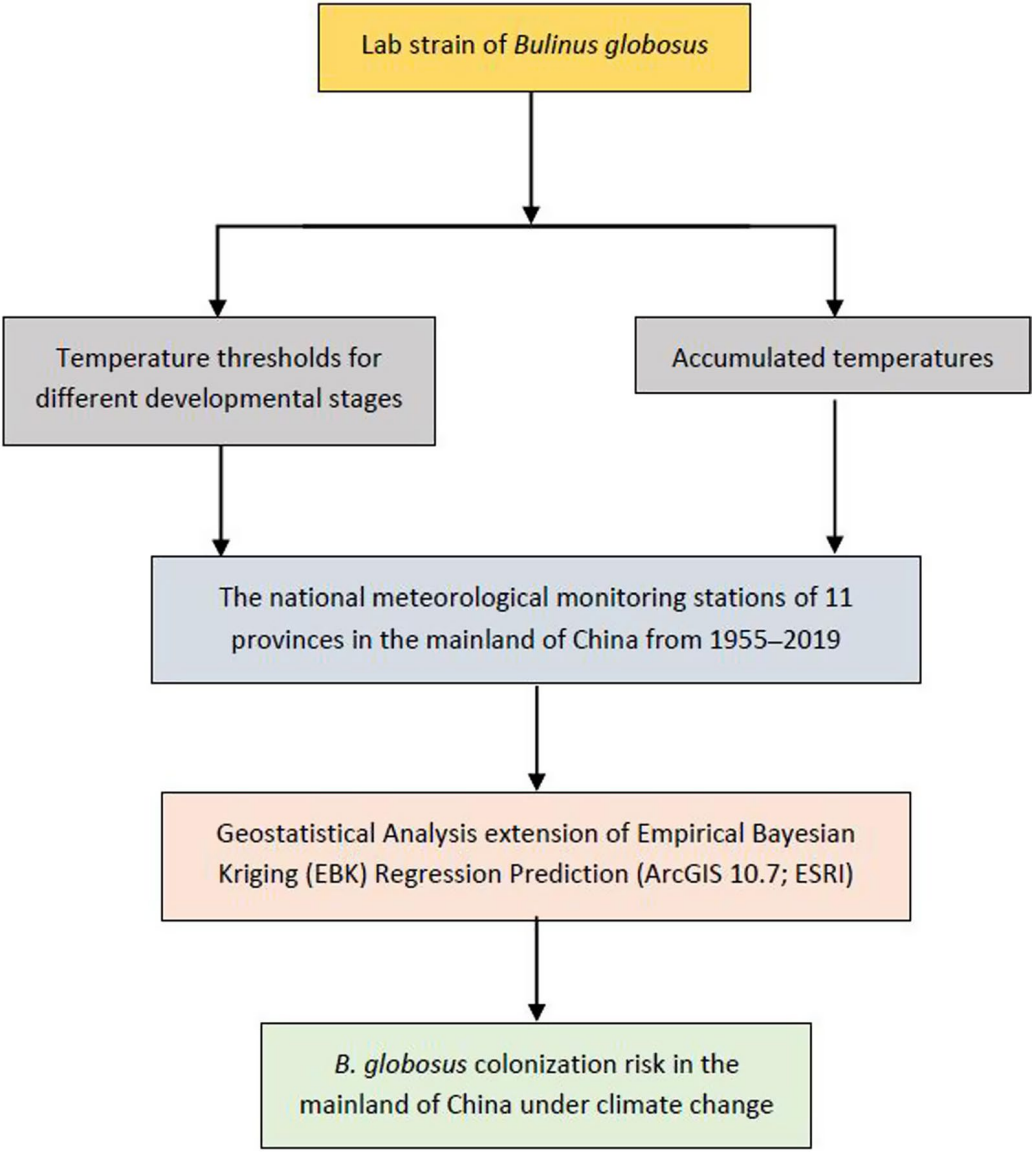
The flow chart of this study process

The aim of this study was to investigate the temperature levels required by the different stages of *B. globosus* snails (eggs, juveniles and adults), as well as for spawning, and to combine these data with those delivered by the national meteorological monitoring stations (MMSs) in southern China. The study is expected to provide a model that can also be used for monitoring the invasion risk of other snails in other countries (Fig. 1).

## Methods

### Snail breeding

*B. globosus* snails, obtained from the laboratory of Sino-African Cooperation for Schistosomiasis Control in Zanzibar, were collected from an on-site pond on Pemba Island where they had been seeded and kept for 3 years. The snails received daylight at 1,000 lx (General environmental experiment box, 12 h light/12 h darkness photoperiod), while kept at 25 °C in 35 × 24 × 20 cm 2-L containers filled with dechlorinated tap water and fed with fresh lettuce every three days. For snail egg collection, several 5 × 5 cm non-toxic plastic films were kept in suspension on the water surface of the breeding containers to act as receptacles. The plastic films were observed daily and when eggs were found, they were scrutinized microscopically and those with intact internal structures were selected for testing. In the experiments, the snail eggs hatched within 2–3 weeks to become juvenile snails that grew into adult snails after another 5–7 weeks.

### Instruments and reagents

The experimental equipment included an MLR-352-PC general environmental experiment box (Panasonic, Osaka, Japan), an ICC50 HD microscope (Leica Camera AG, Wetzlar, Germany), a PolySci 9102 precision temperature water bath (PolyScience, Niles, IL, USA), a MIR-153 biological culture box (Sanyo, Tokyo, Japan), a SB-988 aeration pump (Sobo, Zhongshan Guangdong China). A personal computer equipped with ArcGIS software, v.10.7 (ESRI, Redlands, CA, USA) for geographical information systems (GIS) studies. Dechlorinated water was prepared by adding 0.1 g/L of sodium thiosulfate (Shanghai Aibi Chemical Reagent Co. Shanghai, China) to 50 L of fresh tap water in a plastic tank, stirred until dissolved and left for 12 h before use.

### Temperature thresholds for different developmental stages

As the whole scale of temperatures would not be required for testing, we investigated two limited ranges: one with lower temperatures and another with higher ones to find the thresholds restricting survival. We wished to pinpoint the low and high median effective temperatures (ET50_min_ and ET50_max_) for each stage of snail development (eggs, juveniles and adult snails), i.e., the thresholds at which only 50% of the number of organisms under investigation could survive (or where 50% spawning took place). Each group was set up with 3 replicates, then the mean value of each group is obtained for analysis.

**Fig. 2:**
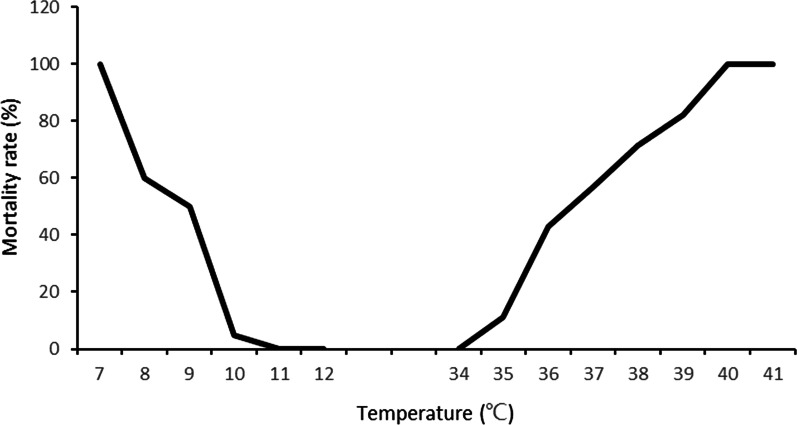
Testing curves of the egg mortality rates at different temperatures

For each group 20‒70 newly deposited snail eggs from aggregated egg collections were investigated by temperature stress testing. The temperature stress testing of snail eggs were divided into 21groups and subjected to different temperatures in experimental water baths containing 10 L of dechlorinated water. The nine low-temperature groups were tested at one-integer increases of temperature from 4.0 °C to 12.0 °C, while the 12 high-temperature groups were tested at temperatures from 34.0 °C to 45.0 °C using the same integer increases. Eggs exposed in different temperatures for 72 h, and then transferred and incubated in a breeding container at 25 °C and observed daily. The hatched eggs in the following 15-day period were counted and those failing to hatch were discarded. The outcome of this experiment revealed the upper and lower thresholds of the range of suitable hatching temperatures. Since the relationship between the mortality rate of the *B. globosus* and temperature is non-linear [19], we used the statistical software SPSS 20.0 (International Business Machines Corporation) to establish the graph (Fig. 2), which is described by the equations governing the changing hatching rate along the change of water temperatures [20, 21]. This gave the ET50_min_ and ET50_max_ for the snail eggs.

**Fig. 3:**
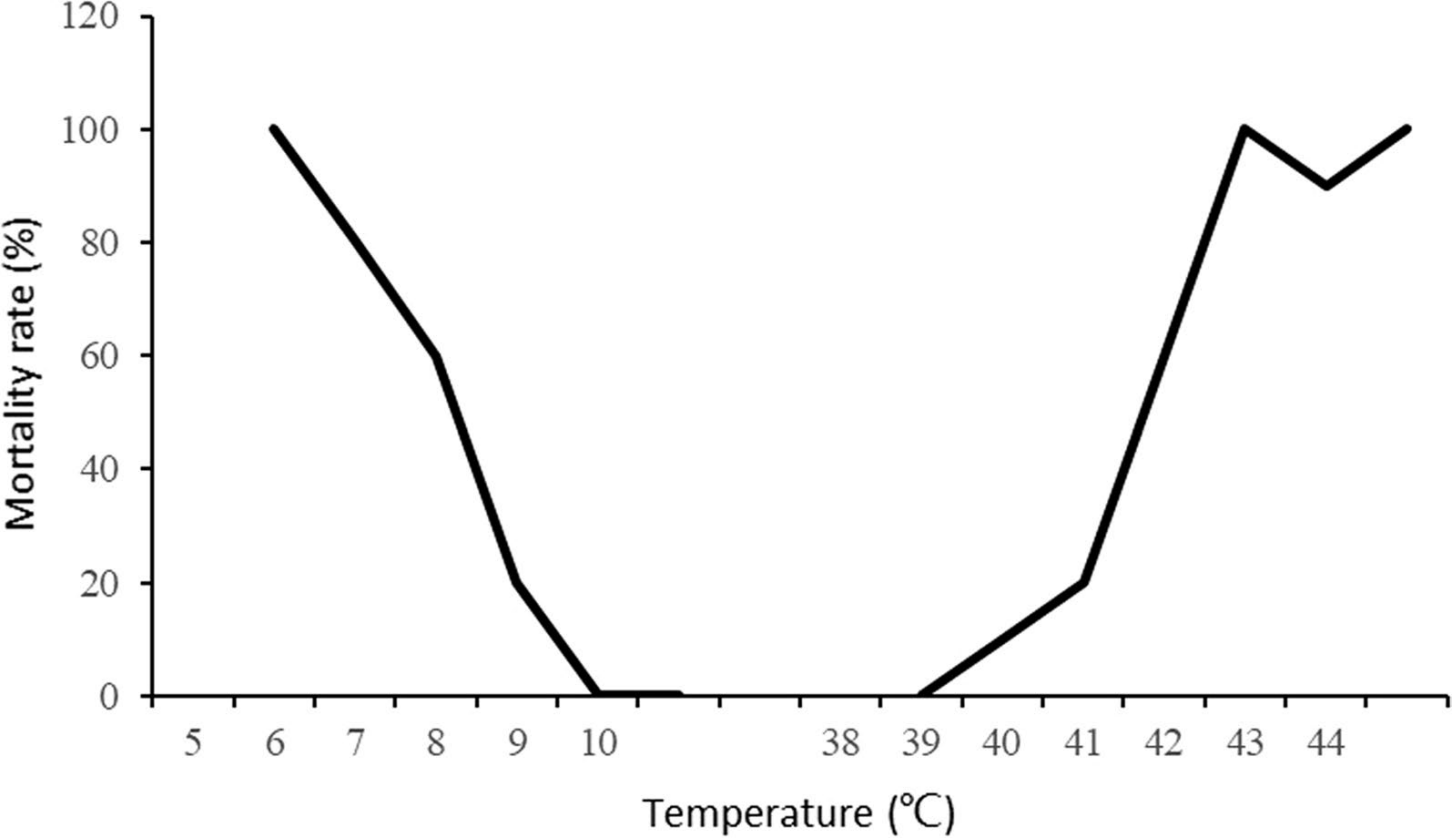
Testing curves of the juvenile mortality rates at different temperatures

**Fig. 4:**
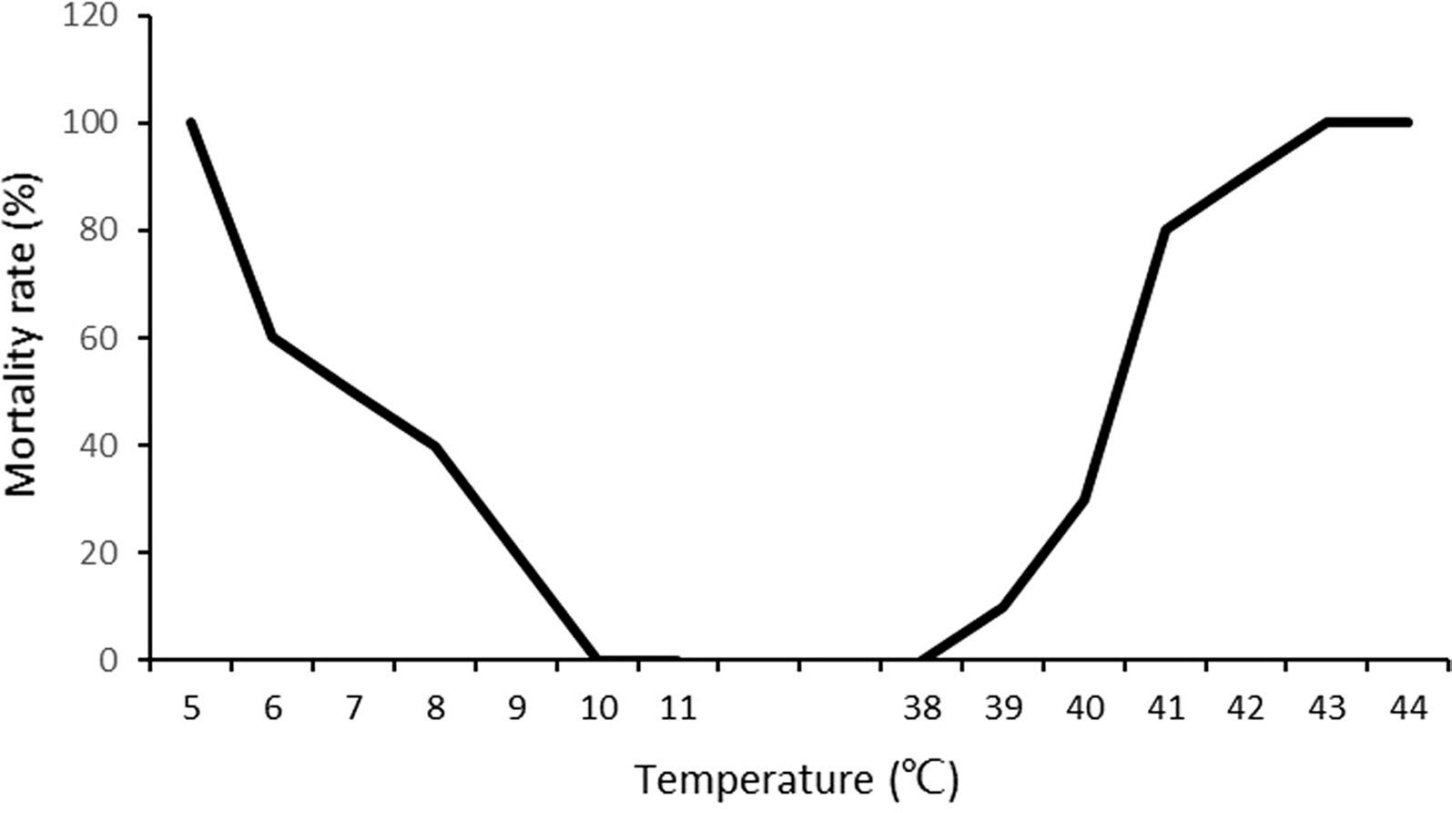
Testing curves of the adult mortality rates at different temperatures

The procedure described above for snail eggs was repeated with 10 juvenile and 10 adult snails in the low-temperature and high-temperature groups with the difference that they were transferred and subjected to 25 °C incubation after 24 h and checked for survival after 72 h using percussion to their head-foot to determine whether they were alive or dead. The ET50_min_ was defined as the minimum water temperature at which the mortality rate reached 50%. The ET50_max_ was defined as the maximum water temperature at which the mortality rate reached 50% (Figs. 3 and 4).

**Fig. 5:**
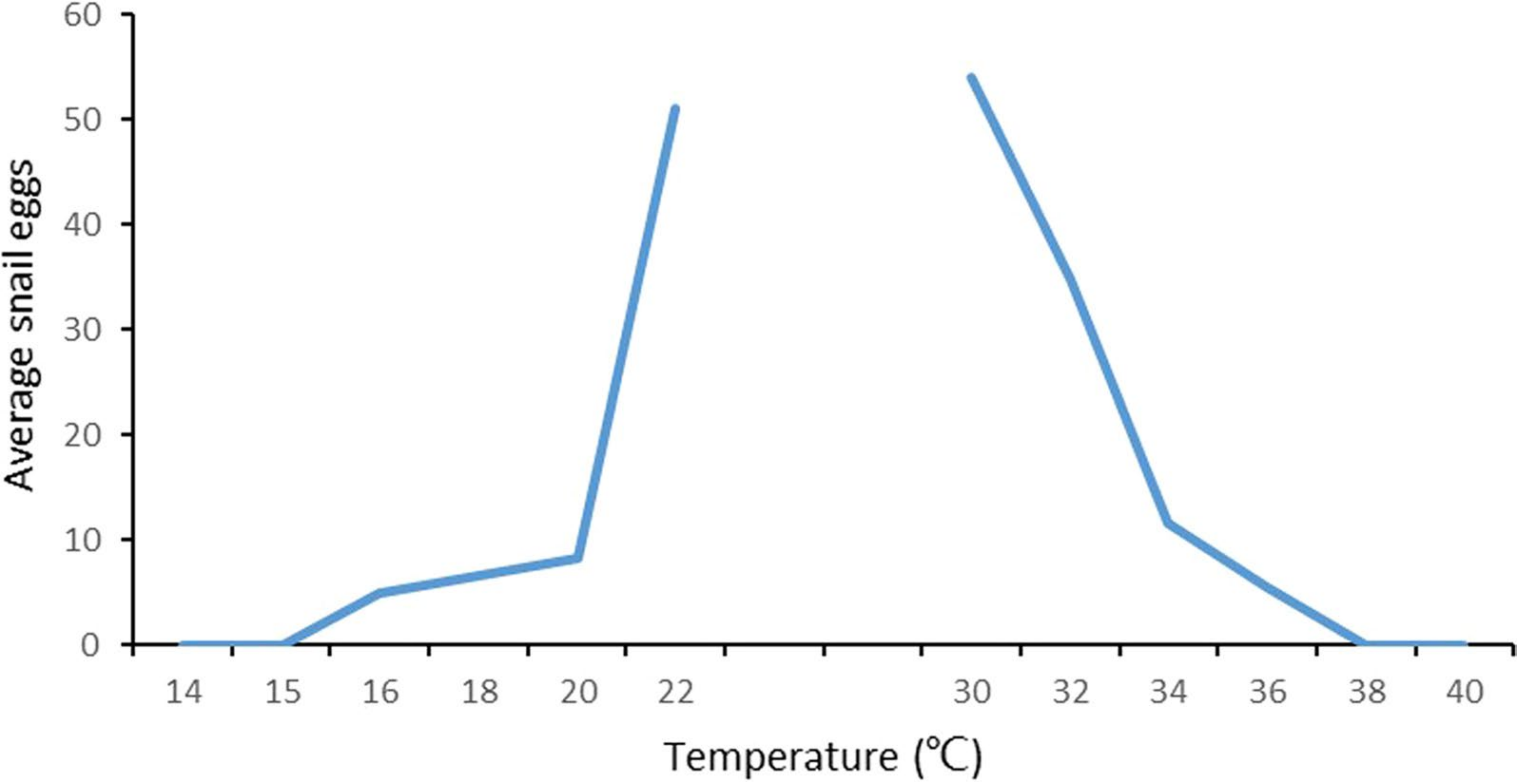
Testing curves of the spawning at different temperatures

Again, 16 groups with different temperatures were compared as described above. This time, however, the water temperature was set to vary from 6.0 °C to 22.0 °C in the water baths in the low-temperature group (6.0, 8.0, 10.0, 12.0, 14.0, 15, 16.0, 18.0, 20.0 and 22.0 °C). The high-temperature group was subjected to vary from 30.0 °C and ending at 40.0 °C (30.0, 32.0, 34.0, 36.0, 38.0 and 40.0 °C). We used three beakers for each temperature group with one adult snail in each, with the snails bred according to the conventional method described above. We observed egg production for each temperature in each beaker and counted the number of eggs over a total of 15 days. Spawning curve equations of the low-temperature group and high-temperature group snails varying with water temperature were established, and the snail spawning extreme at low temperatures and high temperatures were calculated (Fig. 5).

### Accumulated temperatures

To find the temperature needed to keep an unbroken line of snail generations, there was set up with 3 batches at the same time. Between 50 and 100 snail eggs of each batch were bred indoors using terrariums with ample access to water at prevailing ambient temperatures. We observed the whole chain of snail development (eggs, hatching, juveniles, adult snails and spawning) from a temperature–time point of view. The accumulated temperature (AT) from egg deposition to spawning was calculated using the formula:

1 $$AT \, = \, \Sigma \, (T_{DA} - C) \, \left( {T_{DA} > C} \right)$$

where *T*_*DA*_ is the daily average temperature and *C* the threshold temperature of *B. globosus*. *AT* is the product of the prevailing temperature and the number of days counted from egg deposition to the first spawning by the snail originating from the egg (°C d). For a full snail generation to be completed, *T*_*DA*_ must clearly exceed the threshold temperature *C*.

### Calculation of the colonization risk

In order to find the theoretical limits of *B. globosus* in southern China, we obtained a set of meteorological information from the MMSs available from the China Natural Resources Database (http://data.cma.cn/) that contains an inventory of temperatures covering the whole country. We selected the daily average temperatures for the coldest month (January) and the hottest (July) covering the period 1955–2019 from 257 MMSs in the 11 provincial-level administration divisions (PLADs)—Chongqing, Zhejiang, Yunnan, Sichuan, Guizhou, Jiangxi, Hunan, Hainan, Guangdong, Fujian and Guangxi Autonomous Region and compared this information with the 72-h ET50 snail values obtained in the laboratory. The combination of these temperatures would tell us whether or not *B. globosus* would survive to produce consecutive snail generations in China.

We followed the temperatures every ten years to find out which years had recorded temperatures that would have allowed several snail generations. Naturally, we did not get the read-outs from the same number of MMSs each time since their number changed over the long time covered, and the temperature data for the years chosen for the study were not always available. In the years 1955, 1965, 1975, 1985, 1995, 2005, 2015 and 2019, we obtained data from 155, 233, 236, 236, 228, 228, 226 and 197 MMSs, respectively.

As the relationship between temperature and snail mortality [19] could not be expected to be linear, we used SPSS 20.0 software for a variable, non-linear regression analysis based on the T50 measurements discovered to obtain the *B. globosus* colonization risk*.* We calculated the potential, annual generation number (*N*) of *B. globosus* in the various areas by the formula:

2 $$N \, = \, \Sigma \left( {T_{DA} - \, C} \right)/AT$$

where *T*_*DA*_ is the local, average temperature; *C* the starting temperature of snail development (the time of egg deposition); and *AT* the accumulated temperature required for snail development to be completed. As for Eq. 1, *T*_*DA*_ must clearly exceed the threshold temperature for development to occur. Even so, outcomes of *N* < 1 indicate that snails cannot reproduce in the area in question.

### Application of GIS

We used ArcGIS 10.7 software to establish a spatial database of MMS distribution based on geographical coordinates, imported data for survival, reproduction and other attributes as base values for EBK that would generate maps indicating snail survival as well as maps predicting the *B. globosus* colonization risk. The MMSs were established from the founding of the People’s Republic of China in 1949, the data from 1951 to 2019 can be downloaded from the Internet. We generated maps for every 10th year of the study period covering 1955 to 2019 [22]. Superimposing geographical temperature maps of southern China with those depicting the distribution of the temperature thresholds required for survival of the different snail stages, including those needed to allow new snail generations to bridge the year-to-year gap, visualized the changing colonization risk over time, similar to what has been previously shown for agricultural pests in southern China [23, 24].

## Results

### Developmental thresholds of snail

In the 15-day period after 72 h at various temperatures, the egg survival in the low-temperature group was seen to improve along the temperature increases tested. From the 100% mortality rate at 7.0 °C, it reached none (0%) at 11.0 °C with the equation indicating that ET50_min_ were passed at 8.5 °C (Table 1). In the higher end, survival was better than at lower temperatures with 0% mortality rate at 34.0 °C, 100% mortality rate at 40.0 °C and ET50_max_ at 36.6 °C.

**Table 1:** Temperature requirements for the different stages of *B. globosus*

Stage	Temperature range tested, °C	ET50 (°C)	Equation describing the graph	*R*^2^
Egg	T_min_: 4.0–12.0	8.5	Y = 3.929x^2^ − 95.357x + 575.714	0.960
	T_max_: 34.0–45.0	36.6	Y = 1.231x^2^ − 107.492x + 2335.88	0.987
Juvenile	T_min_: 4.0–12.0	7.0	Y = 1.786x^2^ − 49.071x + 305.714	0.965
	T_max_: 34.0–45.0	40.5	Y = − 1.19x^2^ + 116.904x − 2732.845	0.910
Adult	T_min_: 4.0–12.0	7.0	Y = 1.548x^2^ − 40.833x + 260.0	0.967
	T_max_: 34.0–45.0	40.2	Y = − 2.143x^2^ + 195.0x − 4325.715	0.940
Spawning	T_min_: 6.0–22.0	14.9	Y = 1.346x^2^ − 43.202x + 345.006	0.886
	T_max_: 30.0–40.0	38.1	Y = − 0.054x^2^ − 1.57x + 138.097	0.918

The outcomes with respect to the juvenile snails followed this pattern but the survival temperatures were quite different with 100% mortality at 5.0 °C and 0% reached at 8.0 °C with the equation indicating an ET50_min_ of 7.0 °C. At the higher end, the mortality rate was 100%, both at 43.0 °C and 44.0 °C and the ET50_max_ was as high as 40.5 °C. The ET50_min_ and ET50_max_ values of adult snails at 7.0 °C and 40.2 °C, respectively, were very close to those of the juvenile snails. With regard to spawning, the ET50_min_ and ET50_max_ calculated at 14.9 °C and 38.1 °C.

The total development period from egg to adult snail capable of spawning was calculated at 42–65 days (at indoor temperatures varying from 15 °C to 25 °C), with an average of 50.0 ± 5.2 days, while the AT required for completing the development of one snail generation was 607.0–892.3 ℃∙d, with an average of 712.1 ± 64.9 °C∙d.

### The survival and reproductive risk

Calculation of the annual number of snail generations allowed by the temperature records (155, 233, 236, 236, 228, 228, 226 and 197) from each decade’s index year (1955, 1965, 1975, 1985, 1995, 2005, 2015 and 2019), based on the AT values, gave us the permitted number of snail generations per index year investigated (Table 2). Importantly, however, too few development generations cannot meet the colonization needs of the snails, so the low figures in the first columns do not suffice for colonization, while the values given in the following columns do.

**Table 2:** Temporal overview of recorded temperatures 1955–2019 and their impact on snail survival

Year	MMS, *n*	Number and proportion of MMSs having delivered the ATs required to sustain the continuous propagation of snail generations									
		< 1	≥ 1	≥ 2	≥ 3	≥ 4	≥ 5	≥ 6	≥ 7	≥ 8	≥ 9
1955	155	6 3.9%	149 96.1%	143 92.3%	133 85.8%	113 72.9%	66 42.6%	35 22.6%	12 7.7%	1 0.7%	0
1965	233	17 7.3%	216 92.7%	209 89.7%	193 82.8%	166 71.2%	92 39.5%	56 24.0%	25 10.7%	5 2.2%	1 0.4%
1975	236	14 5.9%	222 94.1%	215 91.1%	199 84.3%	169 71.6%	100 42.4%	57 24.2%	22 9.3%	5 2.1%	2 0.9%
1985	236	12 5.8%	224 94.9%	216 91.5%	198 83.9%	169 71.6%	100 42.4%	54 22.9%	19 8.1%	5 2.1%	2 0.9%
1995	228	10 4.4%	218 95.6%	211 92.5%	195 85.5%	170 74.6%	102 20.6%	55 24.1%	22 9.7%	7 3.1%	2 0.9%
2005	228	8 3.5%	220 96.5%	211 92.5%	202 88.6%	180 79.0%	136 60.0%	70 30.7%	38 16.7%	8 3.5%	3 1.3%
2015	226	9 4.0%	217 96.0%	209 92.5%	199 88.1%	182 80.5%	133 58.9%	76 33.6%	44 19.5%	12 5.3%	2 0.9%
2019	197	10 5.1%	187 94.9%	180 91.4%	172 87.3%	152 77.2%	117 59.4%	57 28.9%	33 16.8%	11 5.6%	1 0.5%

*MMS* meteorological monitoring station, *TA* accumulated temperatures

### The northward displacement analysis

**Fig. 6:**
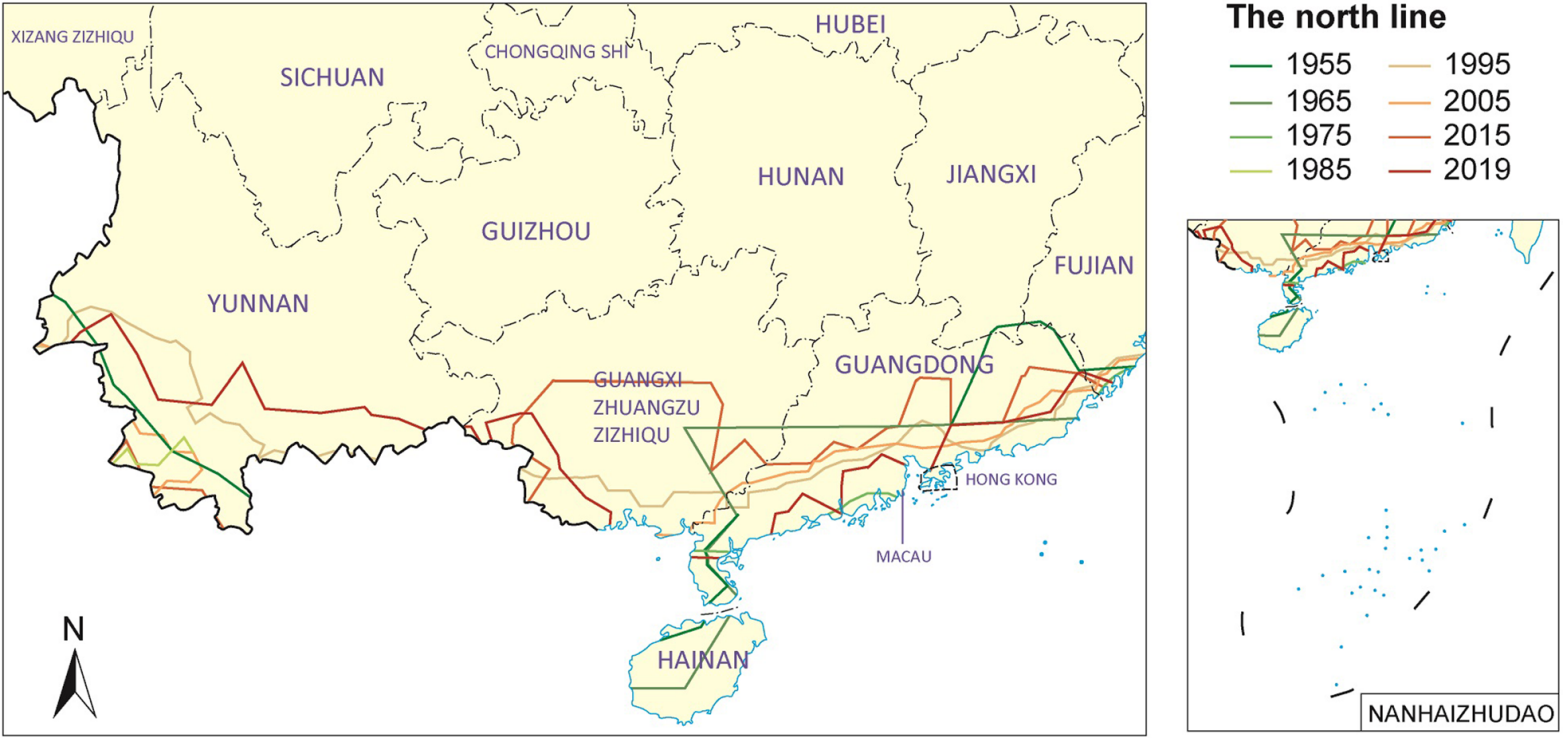
The northward displacement in southern China of the boundary defining areas that could theoretically support colonization of *B. globosus*. Map approval No. GS(2022)2432

The dynamic GIS-based study of the potential distribution of *B. globosus* showed a gradual expansion from South to North during the period 1955–2019. The northern boundary of the potential geographical distribution of snails in 1955 concerned mainly in the coastal areas of Fujian, Guangxi, Guangdong, northern Hainan and Yunnan’s south-western border area gradually (Fig. 2). Importantly, the overall restriction, i.e., the one represented by 2019 of 31.0% (61/197) as it was governed by the egg incubation restriction, a situation mainly seen in Hainan Province and in the southern parts of the provinces of Yunnan, Sichuan, Guizhou, Guangdong, Fujian and Guangxi Autonomous Region (Fig. 6). With respect to the number of snail generations allowed, 59.4% of the MMSs (117/197) reached more than 5 generations and 5.6% of the MMSs (11/197) reached more than 8 generations, which was mainly distributed in the south-eastern area of Yunnan, the southern parts of Guangxi and Guangdong and all of Hainan Province, while only one (0.5% of the MMs or 1/197) reached 9 generations, which indeed was located in Hainan for the year 2019 (Table 2).

### The potential risk of colonization

**Fig. 7:**
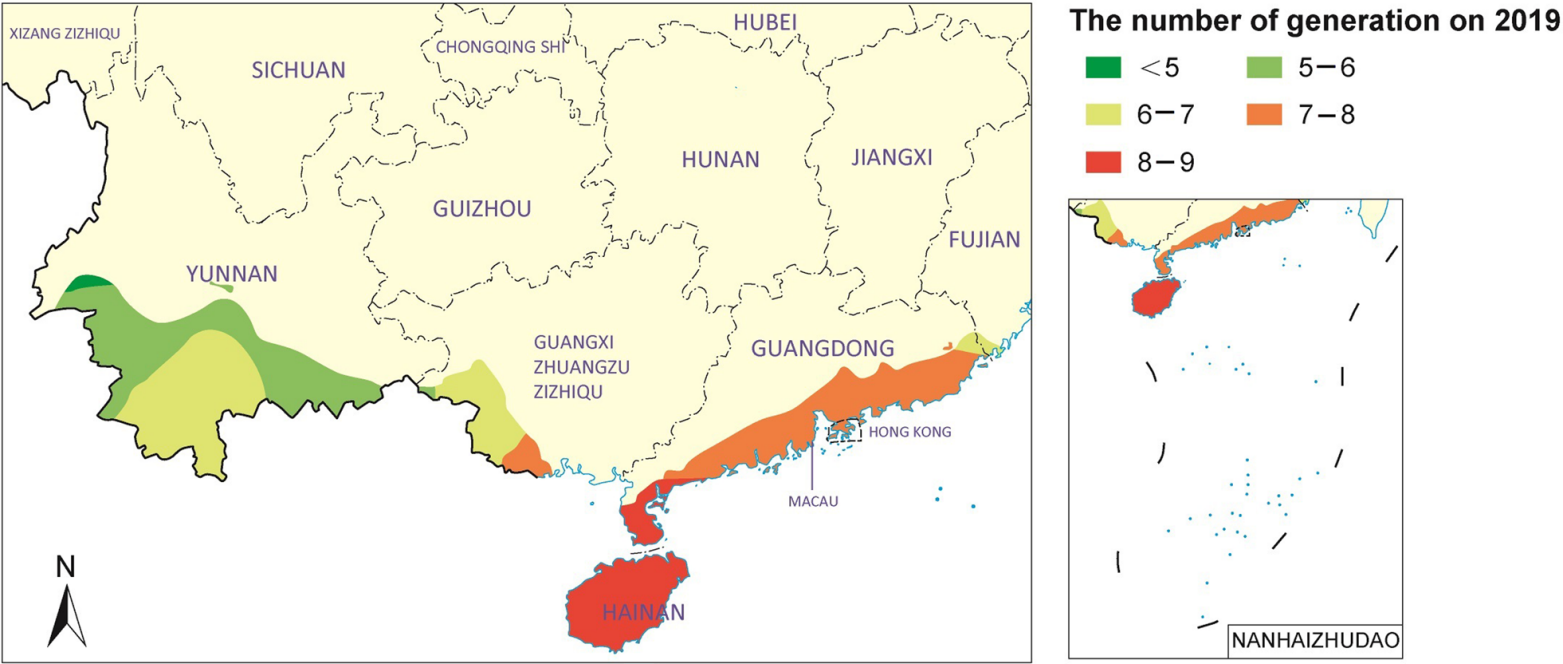
Areas that could theoretically support annual regeneration of *B. globosus* in the mainland of China on 2019. Map approval No. GS(2022)2432

The analysis of the annual generation based on the temperature data from 1955 to 2019 shows that the snail can complete the reproduction of 1–9 generations in more than 90% of the areas monitored by the MMSs of the 11 PLADs in southern China. The distribution area where the number of annual generations was more than 8 showed a gradual expansion trend from South to North, i.e., the number and proportion of MMSs had increased from 1 to 10 (Table 2). This study comprehensively analyzed the superposition of the survival map and the snail annual developmental GIS map for prediction from 1955 to 2019. The areas that could theoretically support annual regeneration of *B. globosus* in the mainland of China is showed a gradual expansion trend from South to North. In 1955, the potential snail distribution would have stretched from the coastal areas of Guangdong Province and Guangxi Autonomous Region to southern Yunnan Province via the borders to Vietnam, Laos and Myanmar. Since then, this line has gradually moved northward and the boundary. In 2019, the areas allowing ≥ 7 snail generations have reached north of the tropic of cancer connecting the southernmost coastal area of Fujian Province with the middle of Yunnan Province via Guangxi and Guangdong. The area meeting the development requirements of 5–9 generations of *B. globosus* is concentrated in parts of Yunnan, Guangdong, Guangxi, Fujian and covers the whole province of Hainan (Fig. 7).

## Discussion

This study depended on China's aid to Zanzibar schistosomiasis prevention and control project that made us investigate the temperature for the growth and development of *B. globosus* snails in the laboratory. We then went on to use the model developed combined with MMS temperature data to find out if these snails could also colonize southern China, as this would involve the potential of schistosomiasis haematobia take hold in China. In this research, we combined ArcGIS 10.7 software with the Geostatistical Analysis extension to use the EBK model to generate maps indicating snail survival as well as maps predicting the *B. globosus* colonization risk. As our findings indicate that this could indeed occur, the government will need to strengthen the supervision of accidental introduction of snails*.*

The relationship between development of organisms and the ambient temperature not only governs the speed of biological development but also sets the boundaries for the distribution of many organisms, particularly those that cannot regulate their body temperature. The AT estimates this time by estimating the amount of heat an organism must accumulate to reach full development. While it must always be within its restricting extreme thresholds, the organism in question obviously needs a longer time at lower temperatures than in a warmer climate. AT was originally used to measure crop maturation, but can also be used for predicting development of parasites, which led to seeing the environment as a set of contiguous niches with diverse ecology as first outlined by Pavlovskii [25] and later developed by Fick and Hijmans [26] into producing the Bioclim environmental records that indicate habitat suitability modelling under past, present or future conditions [27, 28].

Mapping and statistical analysis of climate data contribute to our awareness of how organisms grow and spread. Already in the eighteenth century, René Antoine de Réaumur introduced a unit of AT defined as the amount of heat used an organism needs to accumulate to reach full development [29]. Originally used for predicting the seasonal plant growth in agriculture, this *growing-degree-day* (GDD) unit has more recently been used for widely different measurements, including the development of parasites and also their vectors [30–32]. Here called the AT, this unit accurately expresses the quantitative relationship between snail growth and reproduction and environmental temperature. It is a relatively stable ecological index for the growth and development of snails [33]. The analysis of the annual generation shows that *B. globosus* can complete more than one generation of development and reproduction in 10 PLADs of southern China, which is a potential geographic distribution area. This study comprehensively analyzed the superposition of the survival risk GIS map and the snail annual developmental GIS map for the different index years, one for each decade.

The dynamic GIS-based study of the potential distribution of *B. globosus* showed a gradual expansion from South to North during the study period moving the boundary of the potential geographical distribution of snails from the southern borders and the coastal areas northward. As a reflection of the ongoing global warming of the latest 100 years, which has in the past 30 years pushed the average January temperature in China by 1 °C [32], this potential area includes now large parts of Yunnan, Guangdong, Guangxi as well as the whole province of Hainan and a small area in southern Fujian. Along with the prediction a 0.9 °C rise of the average January temperature in China between 2008 and 2030 followed by another one at 1.6 °C by 2050 [34, 35] and the fact that the minimum temperatures have generally risen, it is obvious that the new temperature levels have already created a more suitable condition for the survival, reproduction and development of *B. globosus,* we face multifaceted risk scenarios. The expansion of the distribution area of snails in general will affect existing geographical schistosomiasis distributions, potentially even lead to the introduction of schistosomiasis haematobium [36, 37].

Although the highest, lowest and average AT have been obtained in the laboratory previously [38–40], we felt that a more detailed investigation is required. We not only found the ET50_max_ and ET50_min_ for each developmental stage of *B. globosus* important but also the thresholds for spawning. The suitable growth temperature for *B. globosus* is 26–29 °C and if the environment temperature exceeds the maximum or minimum temperature for a period of time, it may not survive [41–43]. Knowledge of geographical temperature patterns is very important when attempting to delineate distribution areas and breeding places. Using the ET50 can reduce errors and improve the reliability of the study [44]. The results of this study show very clearly that *B. globosus* do not propagate by eggs deposition at temperatures below 15 °C or above 38 °C (Table 1), which is consistent with the report by Kalinda et al. [19]. The results of the study showed that the range between ET50_min_ and ET50_max_ for *B. globosus* in the laboratory varies between 7.0 °C and 40.5 °C. Thus, from the temperature point of view, the spawning and hatching of the snail egg turned out to be the most vulnerable stage and therefore represent the overall strongest restriction of long-term snail survival.

As *B. globosus* are aquatic snails, the water temperature has a direct impact and sustains the development and reproduction of snails better than air. Since the snails live in shallow waters such as lakes, streams and agricultural irrigation channels, the water and air temperatures are similar. At the same time, there are reports indicating that *B. globosus* is very sensitive to water temperature. For snails living in deep water bodies, when the water temperature in the deep area is lower than the air temperature, the snails will migrate to the surface of the water body or even climb out of the water surface to escape the low temperature. Therefore, this study was carried out in a constant temperature box in the laboratory. To simulate the impact of meteorological temperature on the snail, the temperature data of the model were collected from the national meteorological monitoring point [45–47].

Although there are not yet any signs of *B. globosus* colonization in China, our examination is warranted as *Biomphalaria straminea*, an intermediate snail host of *S. mansoni* in Brazil, was introduced to China in aquatic plants shipments and established habitats in Hong Kong many decades ago. It has not been possible to control that this invasive snail has spread widely in the Pearl River Delta and in Guangdong [48–50]. The risk of the introduction of *B. globosus* into the mainland of China has increased with implementation of “the Belt and Road Initiative” that has opened frequent trade exchange between China and African countries [51]. As the temperature is key for snail survival [52], we focused on this variable leaving other factors, such as light, pH, and oxygen content influence for future studies.

## Conclusions

Our study obtained temperature-related parameters of *B. globosus* through laboratory experiments, and then combined with the meteorological data in the mainland of China to predict the risk of colonization in China via using related models. Our study finds that annual regeneration of *B. globosus* can be supported by the current climate conditions in the mainland of China, and a gradual expansion trend from south to north is shown in the study from 2015 to 2019. Thus, there is a potential risk of colonization of *B. globosus* in the mainland of China under climate change. This study provides a model for monitoring the invasion risk of snails in non-endemic countries. To avoid the incidence and prevalence of schistosomiasis haematobia in China, rigorous control of snail importation and patients with active urogenital schistosomiasis are warranted.

## Data Availability

All data supporting the findings of this study are included in the article and additional file.

## Abbreviations

ET50: Median effective temperatures
AT: Accumulated temperature
GIS: Geographic information system
ET50_min_: Effective minimum temperature
ET50_max_: Corresponding maximum values
IPCC: Intergovernmental panel on climate change
MMSs: Meteorological monitoring stations
GDD: Growing-degree-day
